# Behavioural alterations induced by chronic exposure to 10 nm silicon dioxide nanoparticles

**DOI:** 10.1049/nbt2.12041

**Published:** 2021-03-23

**Authors:** Bashir Jarrar, Amin Al‐Doaiss, Ali Shati, Mohammed Al‐Kahtani, Qais Jarrar

**Affiliations:** ^1^ Nanobiology Unit Department of Biological Sciences College of Science Jerash University Jordan; ^2^ Department of Biology College of Science King Khalid University Saudi Arabia; ^3^ Department of Anatomy and Histology Faculty of Medicine Sana'a University Yemen; ^4^ Department of Applied Pharmaceutical Sciences and Pharmacy Practice Faculty of Pharmacy Isra University Jordan

## Abstract

Silicon dioxide nanoparticles (SiO_2_ NPs) are widely invested in medicine, industry, agriculture, consuming products, optical imaging agents, cosmetics, and drug delivery. However, the toxicity of these NPs on human health and the ecosystem have not been extensively studied and little information is available about their behavioural toxicities. The current study aimed to find out the behavioural alterations that might be induced by chronic exposure to 10 nm SiO_2_ NPs. BALB/C mice were subjected to 36 injections of SiO_2_ NPs (2 mg/kg Bw) and subjected to 11 neurobehavioural tests: elevated plus‐maze test, elevated zero‐maze test, multiradial maze test, open field test, hole‐board test, light‐dark box test, forced swimming test, tail‐suspension test, Morris water‐maze test, Y‐maze test and multiple T‐maze test. Treated mice demonstrated anxiety‐like effect, depression tendency, behavioural despair stress, exploration and locomotors activity reduction with error induction in both reference and working memories. The findings may suggest that silica NPs are anxiogenic and could aggravate depression affecting memory, learning, overall activity and exploratory behaviour. Moreover, the findings may indicate that these nanomaterials (NMs) may induce potential oxidative stress in the body leading to neurobehavioural alterations with possible changes in the vital organ including the central nervous system.

## 1 INTRODUCTION

Silicon dioxide nanoparticles (SiO_2_ NPs) have been extensively invested in various sectors of daily life because of their fascinating physicochemical properties [1, 2, 3, 4]. Nano‐silica are photostable, hydrophobic and hydrophilic nanoforms, inexpensive to produce and easy to prepare. In addition, these nanomaterials (NMs) showed good biocompatibility, significant porosity, and high functionalisation capacity [5]. Silica NPs are invested in drug and gene delivery, cancer treatment, pharmaceutical additives, diagnostic purposes and bio‐imaging [6, 7, 8]. Moreover, SiO_2_ NPs are widely used in agriculture and many industries including sensors, electronics, lubricating oil, paints, textiles, rubber, pigment stabilizers, scratch‐resistance agents and adhesive sealing [5].

Silica NMs are produced in bulky amounts in the global markets and have become the top five NMs used [3, 9]. Research reports indicated that silica NPs may induce toxic effect in human health and the ecosystems because of their unique physicochemical properties [3]. Toxicological findings showed that nano‐silica could accumulate and induce adverse effects in the vital organs specially in the lung, kidney, liver, spleen, smooth muscles and brain with capability of crossing the blood brain barrier [10, 11]. Investigations showed that these NMs could interact with DNA, proteins and lipids with an effect on cancer cell migration and fatty cells differentiation [2, 12, 13]. Some reports showed that SiO_2_ NPs could induce significant alterations in plasma biochemical components and histological alterations in the liver, kidney, lung and testis [14]. In addition, some reports demonstrated that SiO_2_ NPs could cause an inflammatory response, cytotoxicity and cell death with the risk of cardiovascular disease [15, 16, 17]. Moreover, silica NMs were found to alter the immune response and to induce injury to macrophages, erythrocytes and mammalian cell lines together with oxidative stress and nitric oxides disturbance [12, 18].

Silica NMs are considered highly biologically compatible and could accumulate in the corpus striatum [19]. These NMs could cross the intracellular spaces and the placental barrier thereby gaining access to the blood brain barrier [20]. Silica NPs were detected in the placenta, uterus, brain and liver of the offspring when pregnant mice were exposed intravenously to these NMs [11]. In addition, silica NPs are reported to induce homoeostasis disruption, apoptosis and autophagy [21]. Moreover, nano‐silica may induce DNA damage, cell death, oxidative stress and inflammation, with potential neurotoxicity induction [22]. Silica NMs could induce systemic inflammation, reactive oxygen species production, cytotoxicity and autophagy [21, 23, 24]. On the other hand, SiO_2_ NPs significantly lowered DNA synthesis [23]. Moreover, some reports indicated that these nanomaterials could induce cytotoxicity and genotoxicity together with cardiac, pulmonary, hepatic and renal injury [14, 25, 26].

Silica NPs could alter neuromuscular and microgilia function [27, 28]. Studies have proven that silica NPs can cross the blood brain barrier and then access the central nervous system and cause neurotoxicity [24]. These NMs can interact with neurogilia and neurons disturbing the neurological system. Some studies indicated that silica NPs could induce neurotoxicity and enhance neurodegenerative impact on the corpus striatum [22, 24].

Owing to the rapid commercialization and expansive application of silica NMs, concerns are increasing regarding their toxicity on human, organisms and the environment. While some studies reported alterations in some vital organs of the body, the knowledge of SiO_2_ NPs behavioural potential risks may not be well‐established with a need to be addressed. The present study aims to identify the behavioural toxicity that might be produced by chronic exposure to 10 nm SiO_2_ NPs.

## 2 MATERIALS AND METHODS

### 2.1 Silicon dioxide NPs

Spherical and porous uncoated silicon dioxide nanopowder (10 nm, purity of 99.5%, and surface area of 640 m^2^/g) was purchased from Sigma‐Aldrich, USA. Suspension of SiO_2_ NPs was prepared using normal physiological saline (0.9% NaCl) solution. To prevent aggregation, the suspension was sonicated for 15 min before injection.

### 2.2 Experimental animals

A total of 16 adult healthy male BALB/C mice, weighing 25–28 g, were used in the present work. They were divided into two groups: control group and SiO_2_ NPs‐treated one, of eight mice each. All mice were caged with free access to drinking water and pellets diet ad libitum and maintained in an environmentally controlled room (temperature 23 ± 2°C, relative humidity 35% ± 5% and lighting 08:00–16:00 h). Each mouse in the control group received 36 intraperitoneal (ip) injections of 0.2 ml fresh sterilized physiological saline solution, while each member of the SiO_2_ NPs‐treated group received 36 ip injection of SiO_2_ NPs (2 mg/kg Bw). Both groups were injected on the basis of daily single injection for 5 days a week.

General well‐being, mortality, any signs of toxicity and behaviour patterns of all mice under study were observed daily. In addition, mice under study were observed daily for the body position, locomotion, rearing, writhing, lacrimation and alertness reactivity.

### 2.3 Behaviour testing

The behavioural alterations were evaluated 24 h after the latter of the 36 ip injections received. The effect of SiO_2_ NPs on the behaviour of BALB/C mice was evaluated by utilizing 11 standard multiparametric behavioural assays. All animals under study were subjected to neuro‐behavioural tests to evaluate anxiety induction, depression aggravation and the effect on spatial learning, exploring activities and memory in the control and SiO_2_‐treated mice.

#### 2.3.1 Anxiety induction tests

##### The elevated plus‐maze test

**FIGURE 1:**
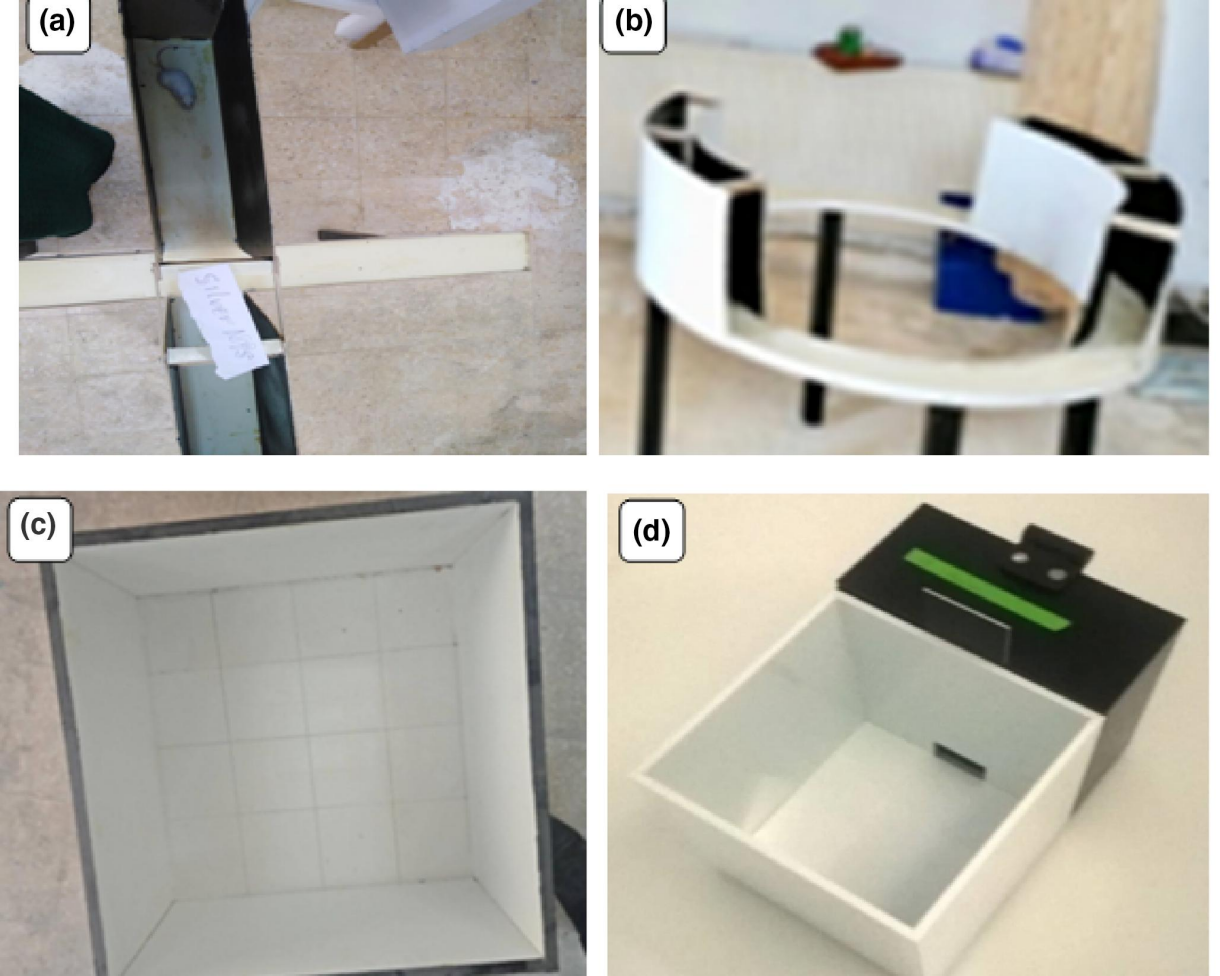
(a)**–**(d)**.** Photographs demonstrate: (a) The elevated plus‐maze is invested in the anxiety‐related behaviour assay. It consists of two crossed arms perpendicular to two open arms and centre area. (b) The zero plus‐maze is employed in anxiety‐like behaviours with two open and two closed areas forming ring‐shaped runway with no central area. (c) The open field maze consists of walled‐platform marked with a grid and squire crossings to evaluate the exploratory behaviour and general locomotor activity levels in relation to anxiety induction. (d) The light‐dark box maze is light/dark transition apparatus employed in the assessment of anxiety responses. This maze is composed of a small dark compartment and a large illuminated one

The apparatus was made of clear wood with opposite arms of 55 cm length, 10 cm width, and 60 cm height from the floor with a central square of side 10 cm each . The closed arms were walled by a 35 cm wall, while the open ones had 2.5 cm height (Figure 1a). The test began with the placement of each mouse individually in the central area, with its head facing the open arm. The total activity was evaluated by recording the time spent in the open arms, the closed arms and the central area. In addition, the number of entries into open and closed arms together with the number of head dipping during a period of 10 min was also recorded. Entry into an arm was considered when the animal placed all the four paws onto the arm. The anxious index was expressed according to Rao and Sadananda [29] as seen in the following equation:

$$\text{Anxious}\;\text{index}\;=\frac{\left(\text{closed}\;\text{arm}\;\text{entries}\;x\;100\right)}{\left(\text{closed}\;\text{arm}\;\text{entries}\;\text{Unsupported}+\;\text{open}\;\text{arm}\;\text{entries}\right)}$$

##### The elevated zero‐maze test

This maze was made of circular platform divided into four areas of equal length and raised 60 cm from the floor while the open quadrants consist of a 10 mm rim. The diameter is 120 cm with a corridor width of 10 and 35 cm height for the dark‐coloured wall (Figure 1b). The total activity was evaluated by recording the time spent in the open areas and the walled ones, number of entries to the open and the walled areas, the number of complete cycles made by the mouse and the number of head dipping [30].

##### The open field test

The used apparatus was manufactured from opaque‐white wooden box (dimensions: 58 cm width × 38 cm height × 40 cm length) with 16 quadrants drawn on the bottom of the box and central square platform in the centre (Figure 1c). Each mouse under study was individually placed on the centre of the field and the video recorded for 15 min. This test based on the principle that the central area is the most threatening to rodents and a line crossing is counted as the mouse passed the line totally [31]. The locomotion within the central quadrants/total locomotion ratio for each mouse was calculated, while the frequency of crossings through the central and peripheral quadrants was used to assess the locomotion activity.

##### The light‐dark box test

The apparatus was made of a small black compartment (20 × 33 × 30 cm) and big white compartment (30 × 33 × 30 cm) separated by a connecting gate (4 × 7 cm) (Figure 1d). Each mouse was placed individually in the centre of the illuminated compartment, facing the opening to the dark compartment. The parameters assessed include time spent in the white and dark compartments and number of entries between the compartments [32, 33]. The activity of each mouse was assessed by the results of the followings: time spent to find the gate, total time spent in the light and dark compartments, number of entries (transitions) and exploring activity (by rearing, head dipping, sniffing and stretch attend posture).

##### The hole‐board test

**FIGURE 2:**
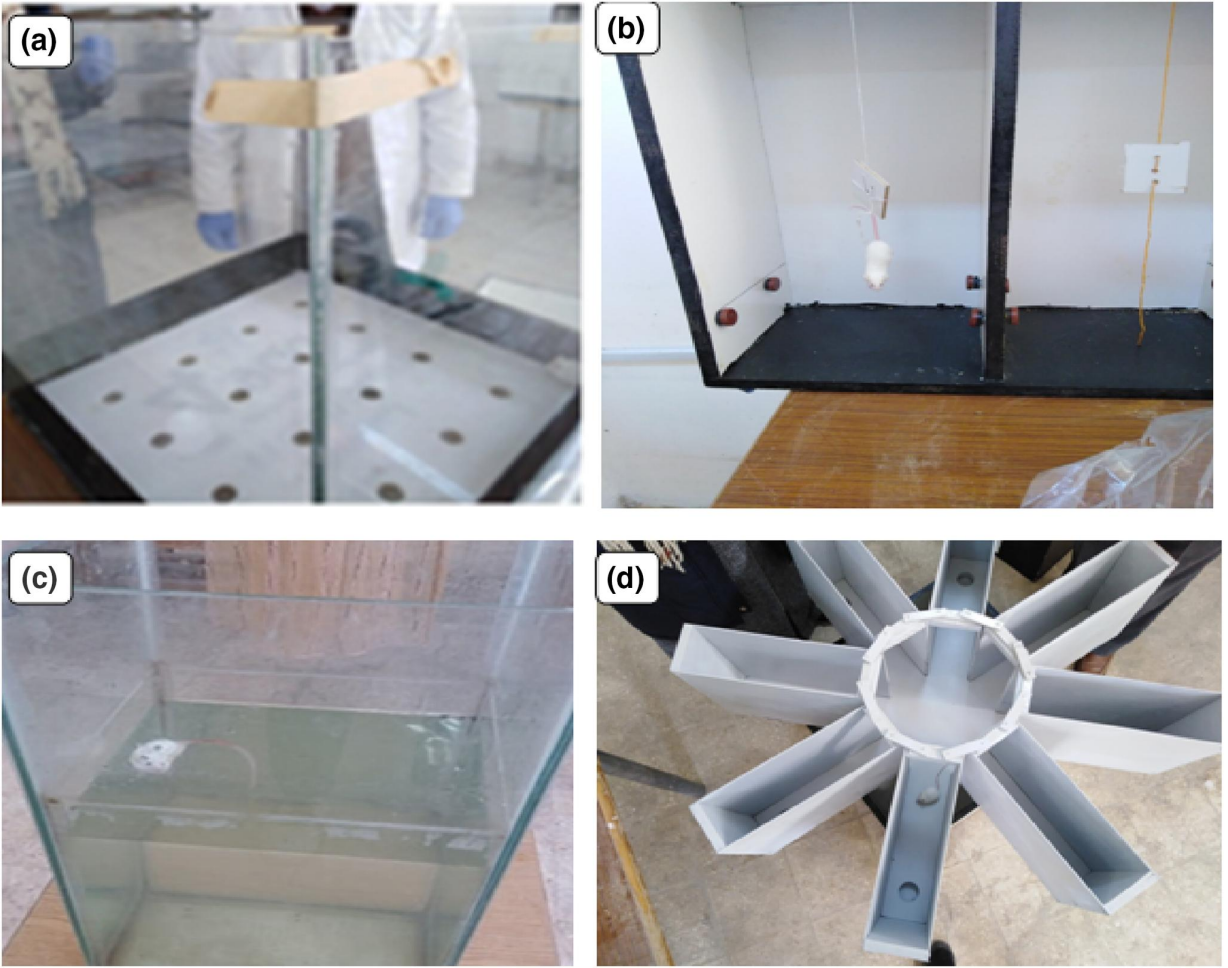
(a)**–**(d)**.** Photographs demonstrate: (a) The hole‐board apparatus is composed of an arena with regularly arranged holes in the floor. This maze is used to assess exploratory behaviour related to anxiety and stresses. (b) The tail suspension maze is used to evaluate the behaviour‐related depression. This apparatus is composed of a large box with a hook hanging down from the top to hang the mouse in a way to hang the mouse without being able to touch the walls or the floor of the apparatus. (c) The forced swimming test is used to assess depression‐like behaviour during swimming with a threat of drowning with no way to escape. This despair apparatus consists of clear transparent cylindrical container filled (2/3) with water. (d) The multiradial maze is employed to assess the working and reference memories performance. This apparatus consists of a central platform and eight spaced arms with invisible food at the end of each arm

This test is used to measure stress, anxiety, neophilia and emotionality in animals. The apparatus was made of a Perspex box (50 × 50 × 40 cm) with the floor covered with 16 holes each with 2.5 cm in diameter (Figure 2a). Each mouse was observed for five exploratory behaviours: thignotaxis, locomotion, head dipping, rearing and central area visiting. Each animal was individually located in the centre of the board for 10 min and observed for these behaviours.

#### 2.3.2 Depression aggravation tests

The following tests were used to evaluate the depressant stress and immobility behaviour trait.

##### The tail suspension test

The apparatus was made of three‐walled rectangular compartment made of wood with the dimensions of (55 cm height ×  60 cm width × 12 cm depth) (Figure 2b). Each mouse was subjected to inescapable stress by suspended it with its tail with tape, in such a position that it cannot escape [34]. The following parameters were assessed: Total time of mobility and that of immobility, number of escapes and rests in the specified time were measured.

##### The forced swimming test

The used apparatus consisted of transparent tank (30 × 30 × 50 cm) filled with water (25 ± 2°C) with water 25 cm from the bottom (Figure 2C). The activity of the mouse was evaluated by the following parameters: total time spent in swimming and immobility, number of rests in the specified time together with the number of climbing attempt [35, 36].

#### 2.3.3 Effects on the spatial learning and memory

The following experiments were conducted to assess the spatial learning performance and spatial navigation memory.

##### The multiradial maze test

Eight radial arm maze apparatus was used in the present work. It consisted of eight horizontal equidistantly spaced arms (50 cm length × 10 cm width × 20 cm height) radiating from circular central area (25 cm in diameter) raised 60 cm from the floor (Figure 2d). The apparatus is used to evaluate the spatial learning and memory in rodents. A food was deposited at the end of each arm. Each food‐deprived mouse was placed on the platform and allowed to move freely for the available food to select among eight arms. After selecting one arm, the mouse returns to the platform before choosing another. An entry was considered every time the mouse placed all paws into the initial entrance of the arm. The reference memory is assessed with the visit to the arms with food, while the working memory is assessed when the mouse made an entry to each arm a single time.

##### Morris water‐maze test

**FIGURE 3:**
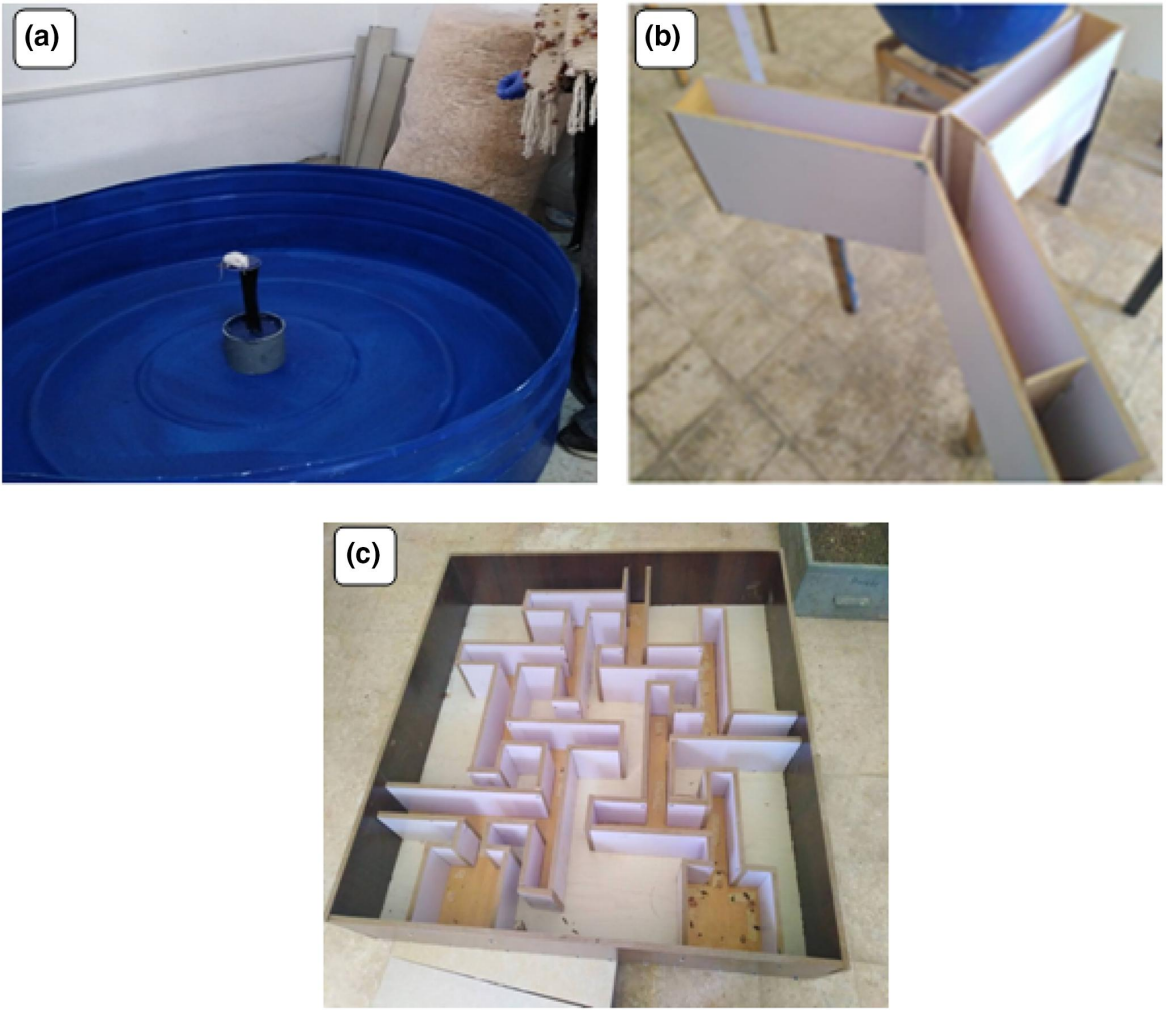
(a)**–**(c)**.** Photographs demonstrate: (a) Morris water‐maze is employed to evaluate the cognition, spatial memory and related forms of learning. This behavioural tank consists of circular water tank with submerged platform with water surface (temperature 22°C) 1 cm below the platform. (b) The Y‐maze test is used to quantify cognitive deficits by measuring the willingness of the mouse to explore a new environment. This Y‐shaped maze has three arms of 120° angles from each other. (c) The multiple T‐maze is used in animal cognition and sequential learning experiments in relation to memory. This apparatus is composed of 12 junctions shaped like the letter T and having the same length and scale

The apparatus was made from a circular pool (height 40 cm, diameter 110 cm) with platform allows the mouse to escape the water. The pool was filled with tap water to a depth of 25 cm (Figure 3a). Each mouse was placed in the pool to find the visible platform. The overall activity was evaluated by the following criteria: escape latency (time to find the platform), time spent in swimming, time spent in immobility and climbing attempts number.

##### The Y‐maze test

The used apparatus has three identical arms (45 cm length, 10 cm width and 20 cm height) shaped like capital ‘Y’ with each arm spaced at an angle of 120° (Figure 3b). Each animal was located individually at the base of the maze and allowed to find the food reward placed in one arm of the maze. The mouse was observed for the followings: side preference, arms alterations, time spent to reach the reward, memory of reward preferences [37].

##### The multiple T‐maze test

This maze was made of 12 identical T‐junctions used to answer questions of places versus response learning and cognitive maps. Each T‐segment consisted of 12 cm wide, 20 cm long and 11 cm high with tracts length from start to the goal segment of 185 cm (Figure 3c). The food‐deprived mouse was placed individually and allowed to explore via the corridors of the maze to find food reward in the last T‐segment, the goal box. The proficiency in approaching the reward indicated that the mouse had generated a cognitive map of the maze during explorations. All mice under study were trained on such procedure three rounds (20 min each) per day for three days. The ability of the hungry mouse to find the food with a decrease in latency and an increase in correct decisions was used to assess the memory retention.

### 2.4 Video recording cameras

Four digital video cameras were used in the present study. The cameras were fixed horizontally and vertically and were supported by a tripod of height enough for resolution to produce high quality images.

Each of the used apparatuses and tools were wiped thoroughly after each session for each mouse with a 70% alcohol solution between test sessions. Moreover, all animals under study were treated according to the National Institute of Health Guide for the care and use of the mice under study.

### 2.5 Data analyses

Statistical data analysis was carried out using one‐way ANOVA. The findings were expressed as the average ± SD. The *p*‐value < 0.05 was considered statistically significant. The data were analysed by SPSS software.

## 3 RESULTS AND DISCUSSION

The control mice and those exposed to SiO_2_ NPs did not present any clinical signs of toxicity with no mortality that was recorded among SiO_2_ NPs‐treated mice. The followings are the behavioural alterations demonstrated by mice received 36 ip injection of SiO_2_ NPs (2 mg/kg Bw) in comparison with the control ones.

### 3.1 The elevated plus‐maze test

**FIGURE 4:**
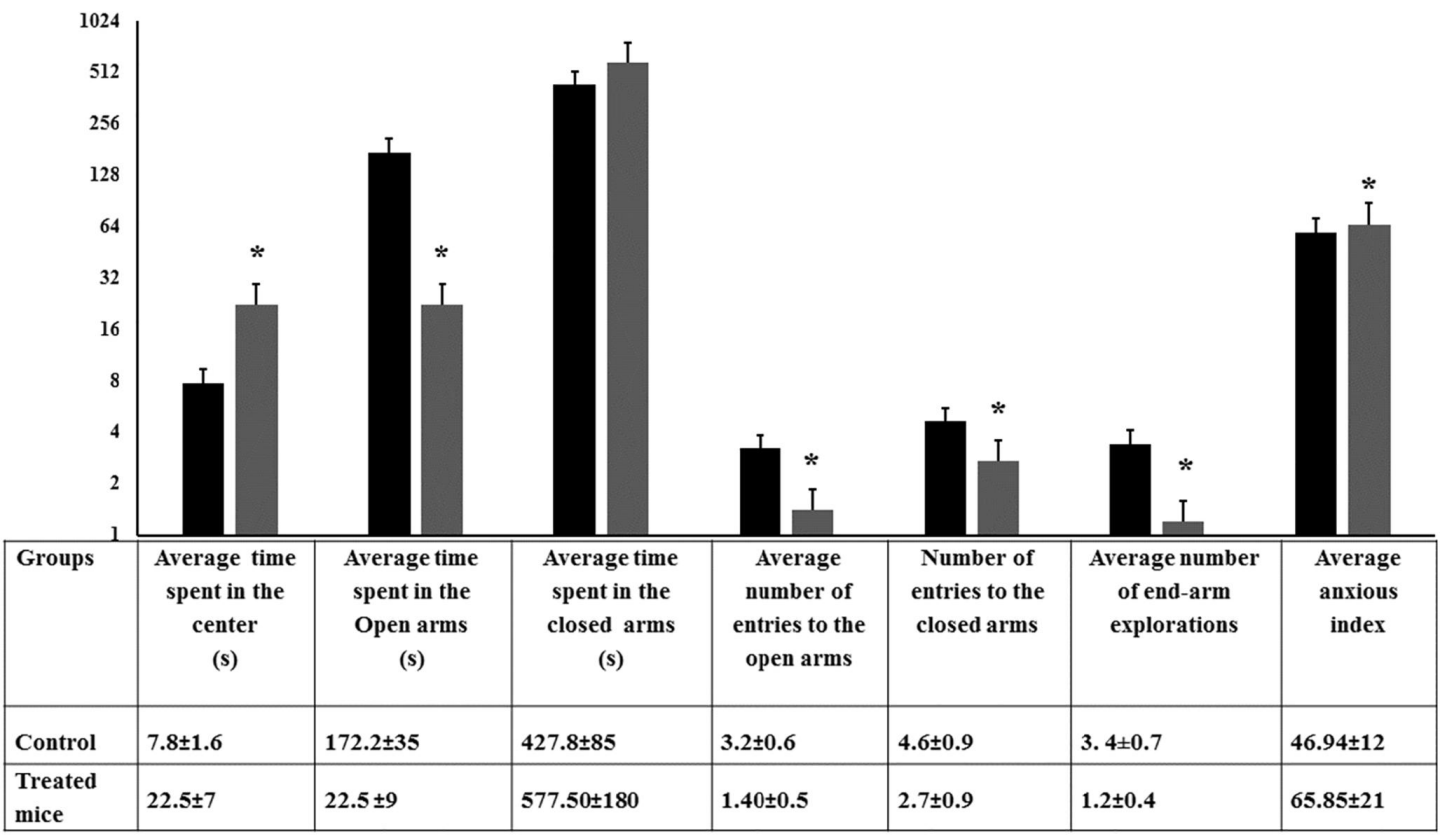
Behavioural performance of control and SiO_2_ NPs‐treated mice in the elevated plus‐maze test. The time spent in the open arms, closed arms, and central area of the maze together with number of entries and head dipping during a period of 10 min were recorded for the mice under evaluation. ‘*’ indicates statistical significance using one‐way ANOVA test and *p* value < 0.05, compared with the control group. Black bar: Control mice; Gray bar: SiO_2_ NPs‐treated mice

This test is an aetiologically valid animal model of anxiety used as a natural fear stimuli of an open space. An anxiety‐induced agent decreases the time spent in open arms and increases the time spent in enclosed arms [30]. In the present test, the anxiety was expressed by the time spent in the enclosed arms. Compared to the control animals as seen in Figure 4, exposure to nano‐silica induced anxiety‐like effect in the treated mice, as it significantly lowered the time spent in open arms and elevated the time spent in the closed arms. In addition, SiO_2_ NPs‐treated mice dampened the number of entries to the arms and the number of head dipping. These together may indicate anxiogenic effect of silica NMs. In addition, SiO_2_ NPs‐treated mice significantly elevated the anxious index in comparison with the control animals (65.84 ± 21 vs. 46.94 ± 12).

### 3.2 The elevated zero‐maze test

This test is used to assess behaviour related to anxiety in rodents, based on exploring a novel environment and avoiding open spaces constituting situations of predator risk [30]. Shorter time intervals spent in the open areas are interpreted as increased anxiety while the number of entries into closed compartment is an index of general activity**.**

**FIGURE 5:**
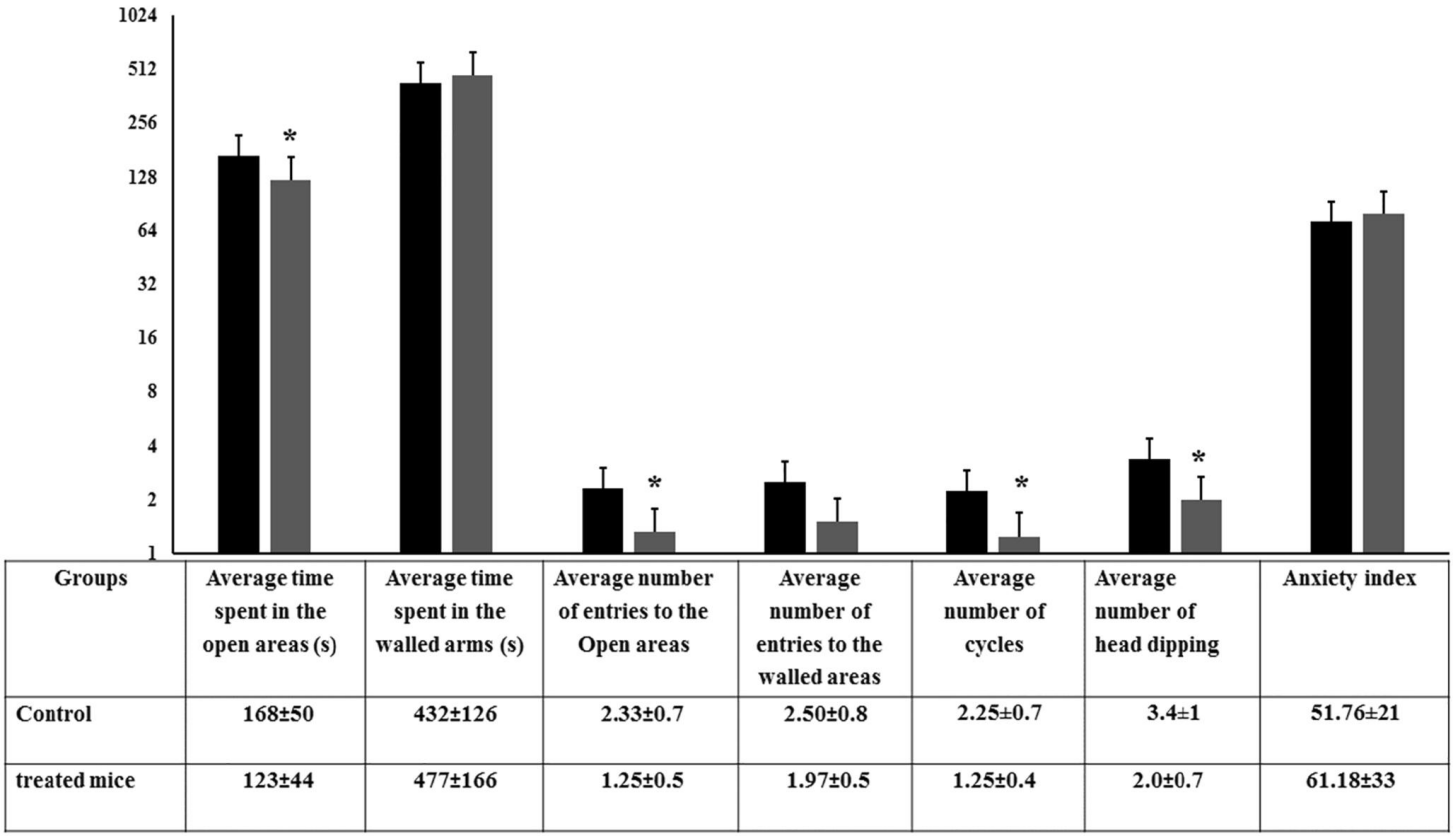
Behavioural performance of control and SiO_2_ NPs‐treated mice in the elevated zero‐maze test. The time spent in the open and the walled areas, number of entries to the open and the walled areas, the number of complete cycles and the number of head dipping made by each mouse under study were recorded. In addition, the histogram shows the anxiety index of the control and silica NPs‐treated mice ‘*’ indicates statistical significance using one‐way ANOVA test and *p* value < 0.05, compared with the control group. Black bar: Control mice; Gray bar: SiO_2_ NPs‐treated mice

As seen in Figure 5, exposure to nano‐silica decreased significantly the average time spent in the dark areas, the numbers of entries, head dipping and over all activity. It has been reported that increased anxiety and/or fear level is related to a decreased exploration behavioural patterns and serotonergic system activity reduction [24].

### 3.3 The open field test

**FIGURE 6:**
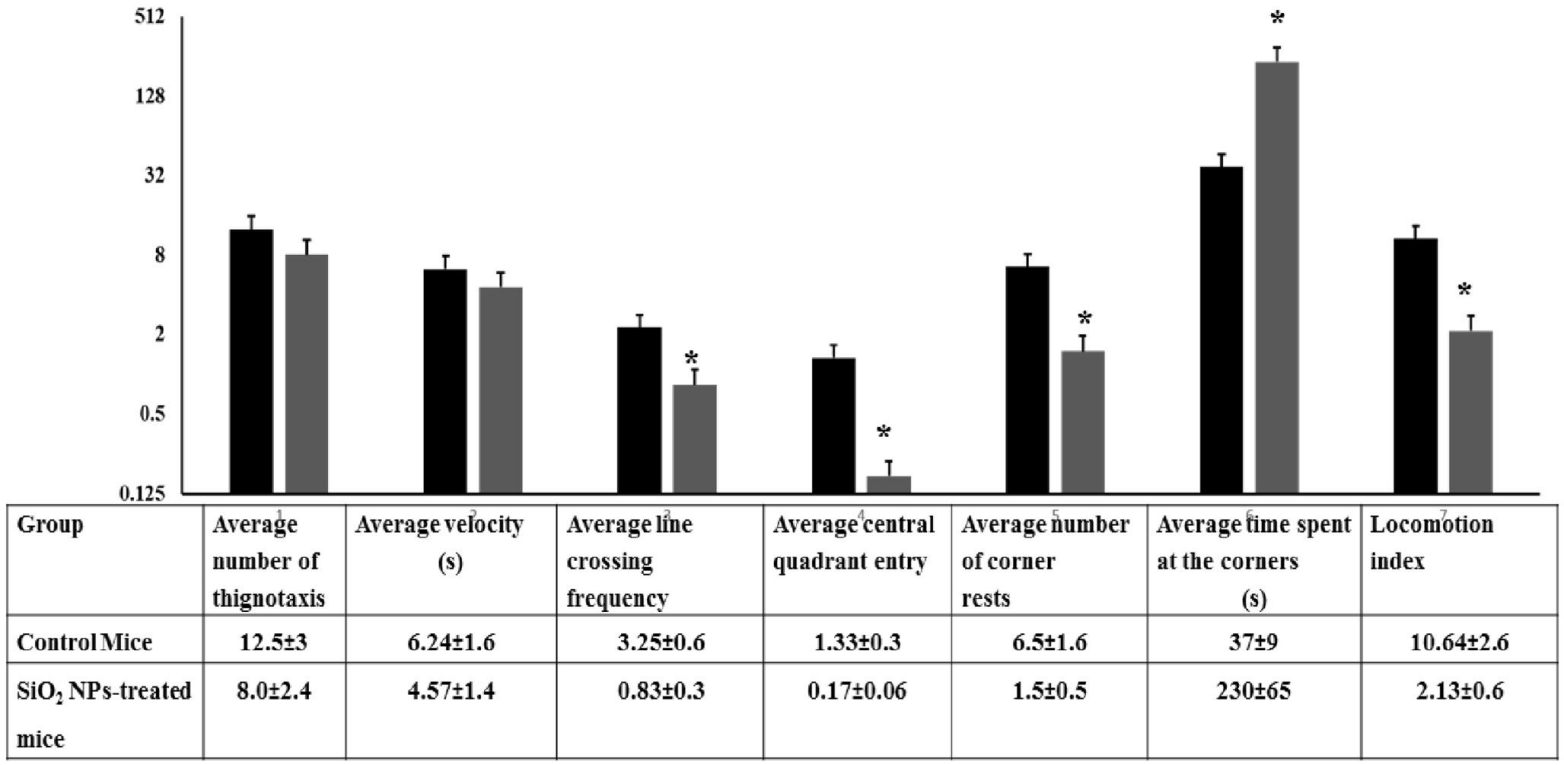
Behavioural performance of control and SiO_2_ NPs‐treated mice in the open field test (*T* = 15 min). Each mouse was video recorded for the number of thignotaxis, the locomotion within the central quadrants, number of corner rests and the crossing frequency through the central and peripheral quadrants. The present histogram demonstrates the locomotion velocity and locomotion index of the mice under study. ‘*’ indicates statistical significance using one‐way ANOVA test and *p* value < 0.05, compared with the control group. Black bar: Control mice; Gray bar: SiO_2_ NPs‐treated mice

This test investigates anxiety induction, exploration and locomotion alterations [31]. Anxiety is indicated by lowering the total locomotors activity, distance travelled in central quadrant and time (%) spent in central area, with elevation the time spent in the periphery zone and in the apparatus corners [38]. Figure 6 demonstrates thignotaxis activity, line crossing frequency, central quadrant entry (transition), number of rests at the corners and the time spent at the corners. It is obvious from the findings that subjection to silica NPs reduced the thignotaxis activity and line crossing frequency with suppression to the central quadrant entry. Moreover, SiO_2_ NPs increased the average number of corner rests but significantly decreased the locomotion index compared with the control mice (2.13 ± 0.6 versus. 10.64 ± 2.6.

### 3.4 The light‐dark box test

**FIGURE 7:**
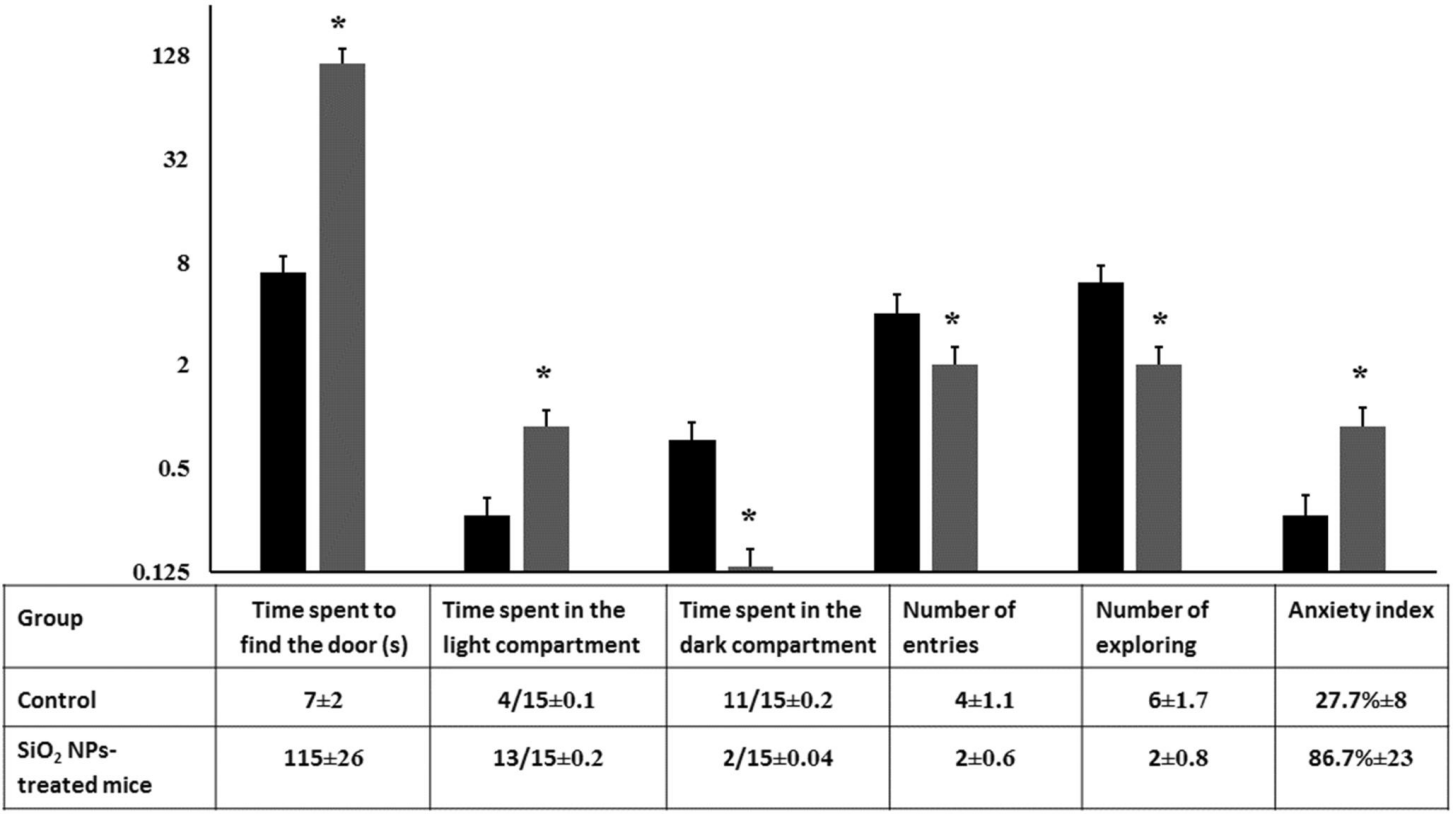
Behavioural performance of control and SiO_2_ NPs‐treated mice in the light‐dark box test (*T* = 15 min). Each mouse was observed for the time spent to find the gate, total time spent in the light and dark compartments, number of entries and exploring activities. The histogram shows the anxiety index for the control and silica‐treated mice. ‘*’ indicates statistical significance using one‐way ANOVA test and *p* value < 0.05, compared with the control group. Black bar: Control mice; Gray bar: SiO_2_ NPs‐treated mice

This behavioural paradigm test is an experimental procedure used to test anxiety in laboratory animals. Rodents prefer darker area than the lighter one and when presented in a novel environment, they have a tendency to explore [33]. As seen in Figure 7, exposure to SiO_2_ NPs increased the time spent in the light compartment the time spent to find the door and the anxious index but decreased the time spent in the dark compartment, number of entries to the dark compartment and the number of exploring. These together may indicate that SiO_2_ treated‐mice are more anxious than the control ones and silica NMs are anxiogenic agents with the ability to induce anxiety‐like behaviour and reduce locomotor activity [32].

### 3.5 The hole‐board test

**FIGURE 8:**
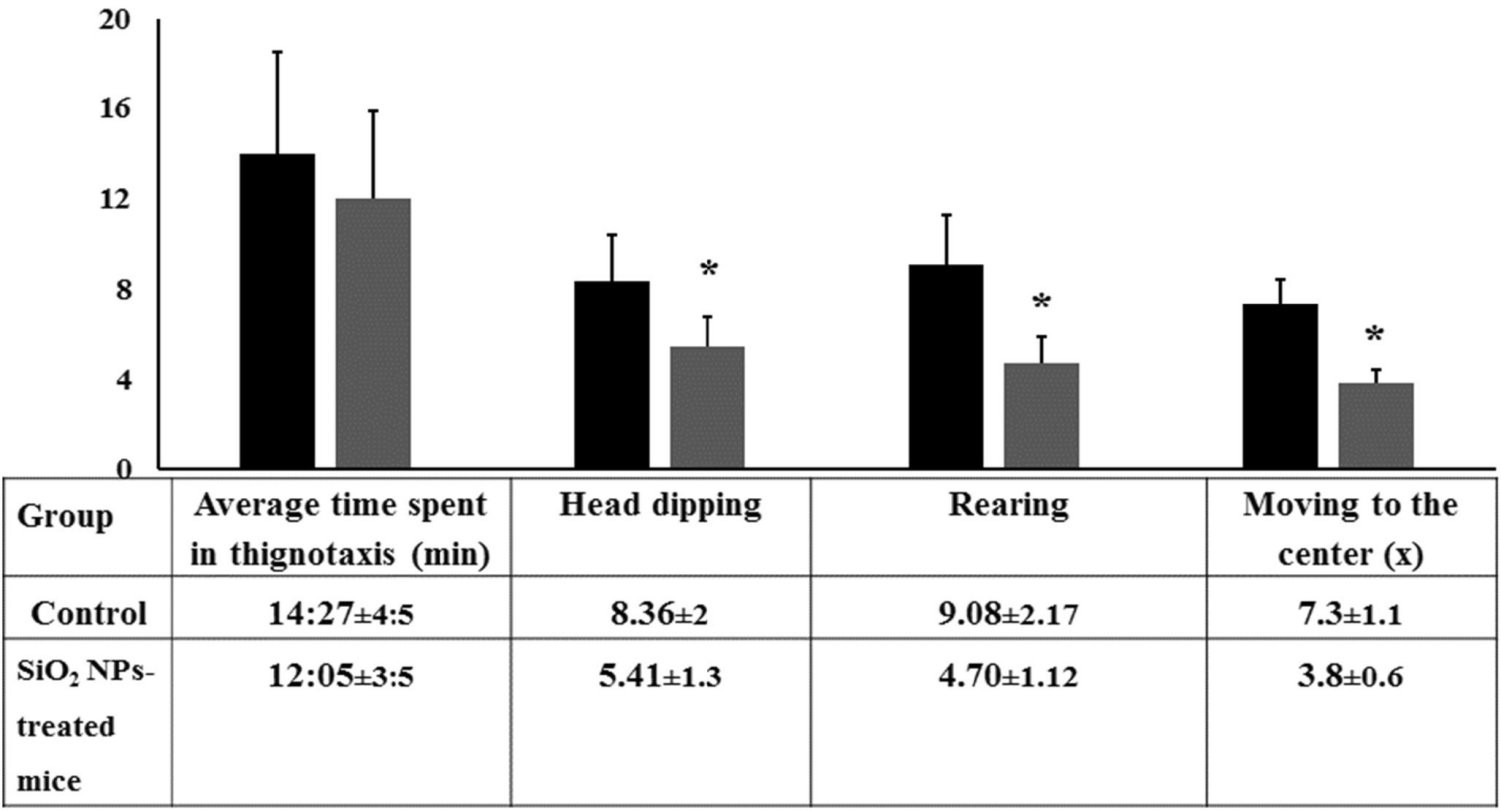
Behavioural performance of control and SiO_2_ NPs‐treated mice in the hole‐board test (*T* = 10 min). Each mouse under study was observed for thignotaxis, head dipping, rearing and central area visiting. ‘*’ indicates statistical significance using one‐way ANOVA test and *p* value < 0.05, compared with the control group. Black bar: Control mice; Grey bar: SiO_2_ NPs‐treated mice

This experimental test is used to measure multiple emotionality behaviours in rodents mainly anxiety and exploratory behaviour [39]. Head dipping, rearing, and locomotion indicate the common activities. The more these exploratory behaviours occur, the less anxious the animal is and if the animal does not show these behaviours then it is more anxious**.** As seen in Figure 8, exposure to silica NPs lowered the activity and exploration where SiO_2_‐treated mice demonstrated reduction in numbers of head dipping and rearing in comparison with the control ones. By entering a new place, exploration provides the animal with information about its novelty of having food, shelter or mating partners with a risk of predation and aggression from various hazards. In addition, subjection to nano‐silica lowered thignotaxis and locomotion rate. These results may reveal that silica NMs can induce stress leading to a decrease in neophilia towards neophobia with fear‐based avoidance stressing the object conflict avoidance [39].

### 3.6 The tail‐suspension test

**FIGURE 9:**
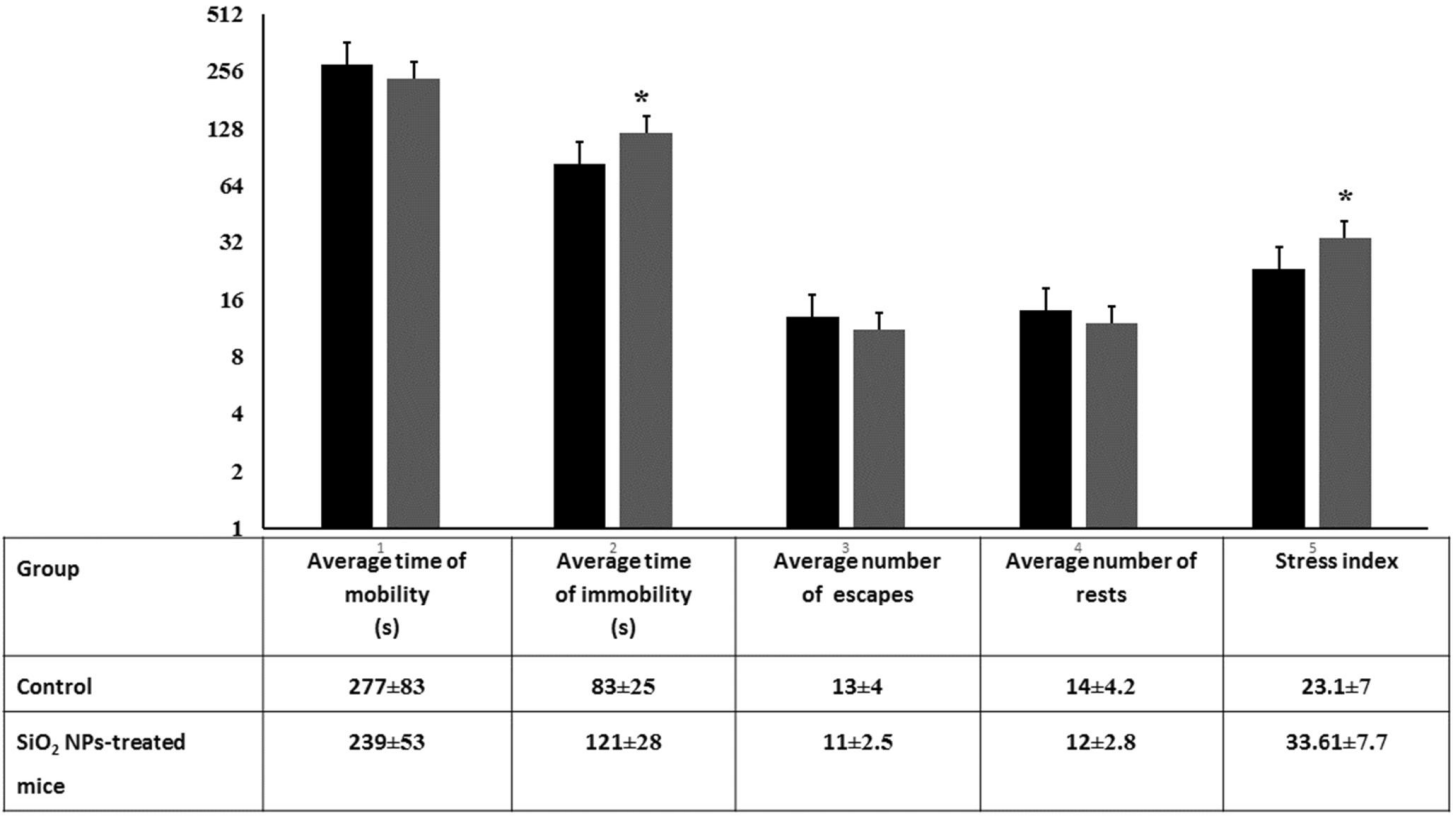
Behavioural performance of control and SiO_2_ NPs‐treated mice in the tail suspension test. Each mouse was subjected to inescapable stress on suspending it with its tail and assessed for the total time of mobility and immobility, number of escapes and rests in the specified time (6 min). The histogram shows the calculated stress index for the control and treated mice. ‘*’ indicates statistical significance using one‐way ANOVA test and *p* value < 0.05, compared with the control group. Black bar: Control mice; Gray bar: SiO_2_ NPs‐treated mice

This behavioural test is invested to measure stress in some experimental animals including mice [34]. The time it took until each mouse under study remained immobile and the stress index were measured**.** As seen in Figure 9, SiO_2_ NPs exhibited increased immobility and stress index. This finding may indicate that subjection to SiO_2_ NPs may aggravate insightful depression that putting the animal in a position of defeat with the ambient environment demonstrating depression‐like behaviour.

### 3.7 The forced swimming test

**FIGURE 10:**
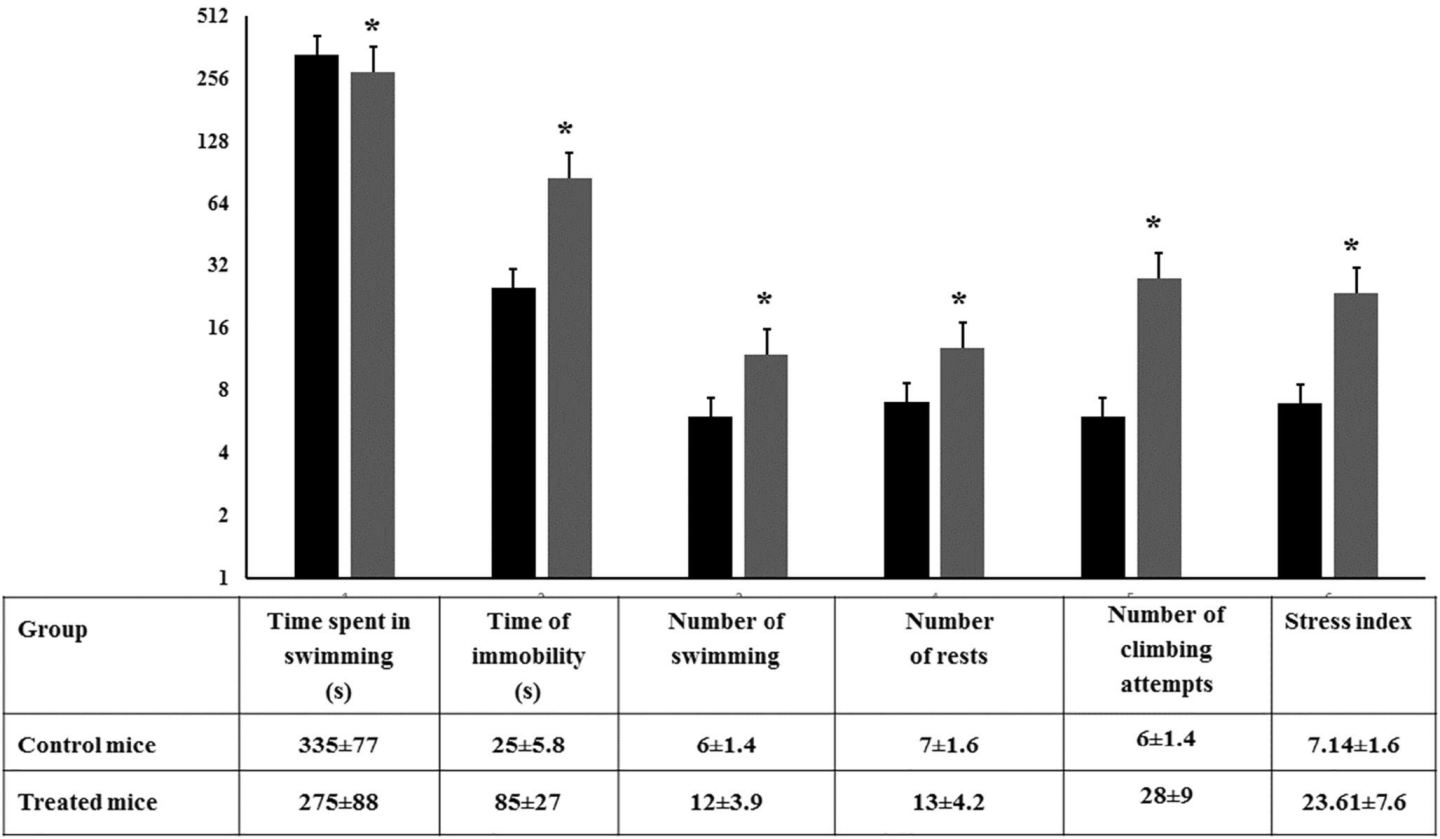
Behavioural performance of control and SiO_2_ NPs‐treated mice in the forced swimming test (*T* = 6 min). Each mouse under study was evaluated by the total time spent in swimming and immobility, number of swimming and rests, and the number of climbing attempts. In addition, the histogram shows the calculated stress index for each group of animals. ‘*’ indicates statistical significance using one‐way ANOVA test and *p* value < 0.05, compared with the control group. Black bar: Control mice; Gray bar: SiO_2_ NPs‐treated mice

This test is used to evaluate depression induction [35, 40]. When the mouse is placed in a transparent container filled with water, it will struggle to escape but eventually will demonstrate immobility due to behavioural despair stress which may indicate the tendency for major depression. In theory, a depressed mouse gives up more quickly than a happy one. As seen in Figure 10, SiO_2_ NPs‐treated mice demonstrated an increase in immobility and high stress index. Depression is accompanied with stress and a lack of ability to handle and associated with many disorders, including, metabolic, cardiovascular and neuropsychiatric disorders [36, 41, 42].

### 3.8 The multiradial maze test

This test measures the spatial learning and memory in rodents. The reference memory is assessed with mouse's visit to the arms with food. The failure to do so indicates an error in the reference memory while the working memory is assessed when the mouse made an entry to each arm a single time. Re‐entry into the arms with no food reward would be considered as working memory error [43].

**FIGURE 11:**
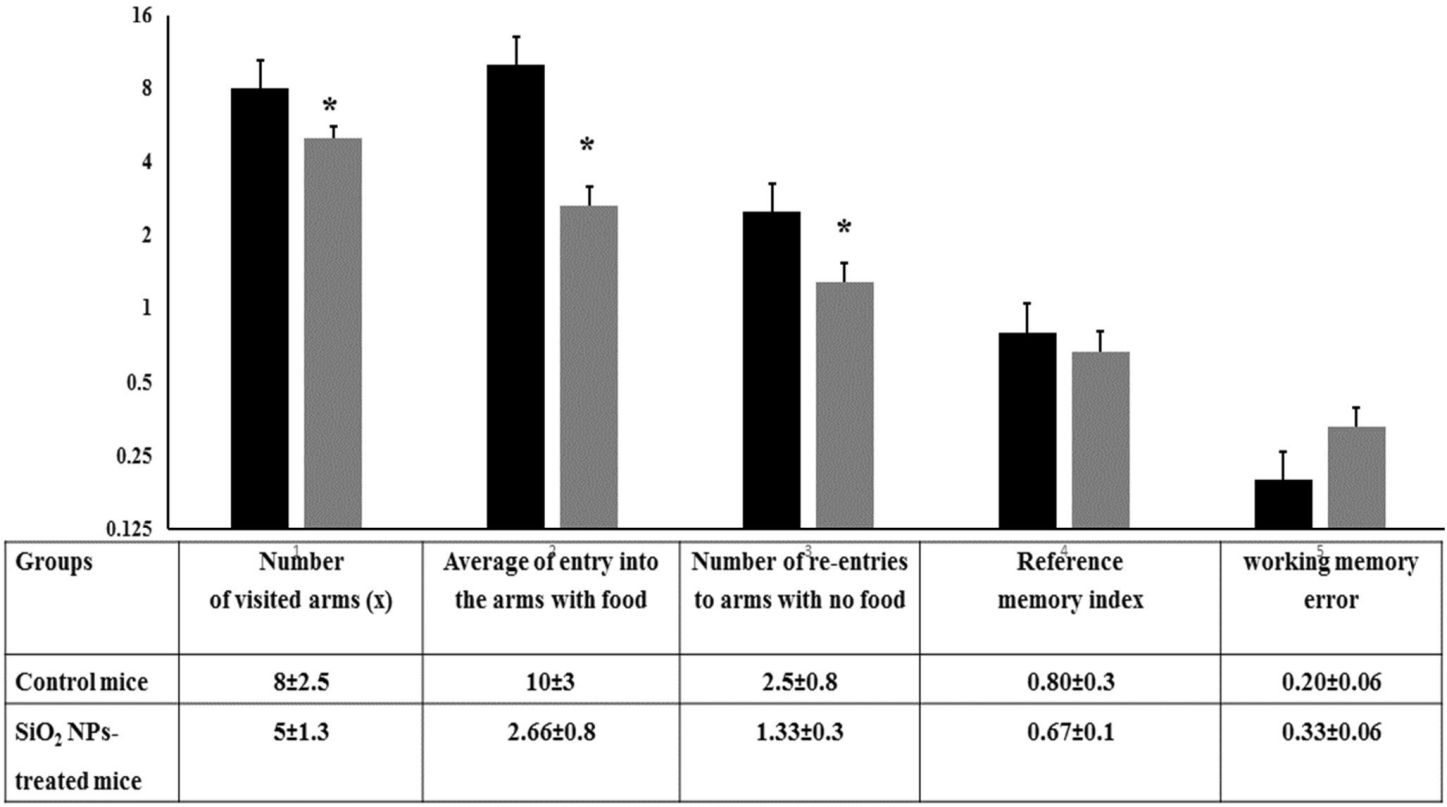
Behavioural performance of control and SiO_2_ NPs‐treated mice in the multiradial maze test (*T* = 10 min). Each food‐deprived mouse was placed on the platform and allowed to move freely for the available food to select among eight arms before was being returned to the platform to choose another. The reference memory and the working memory were assessed for the control and the treated mice. ‘*’ indicates statistical significance using one‐way ANOVA test and *p* value < 0.05, compared with the control group. Black bar: Control mice; Gray bar: SiO_2_ NPs‐treated mice

The overall activity was assessed by the total number of entries and re‐entries to all arms as seen in Figure 11. Silica NPs treated mice demonstrated reduction in the number of entries into the arms with food in comparison with the control mice. In addition, treated‐mice demonstrated higher working memory error and lower reference memory index. The result of the present behavioural test indicates that exposure to silica NMs affect both the reference memory and the working memory. Survival in new environment depends on learning of remembering locations [44]. The results of the present behavioural test may indicate that exposure to SiO_2_ NPs lowered the capacity of the mouse to manoeuvre safely in entering new location.

### 3.9 Morris water‐maze test

**FIGURE 12:**
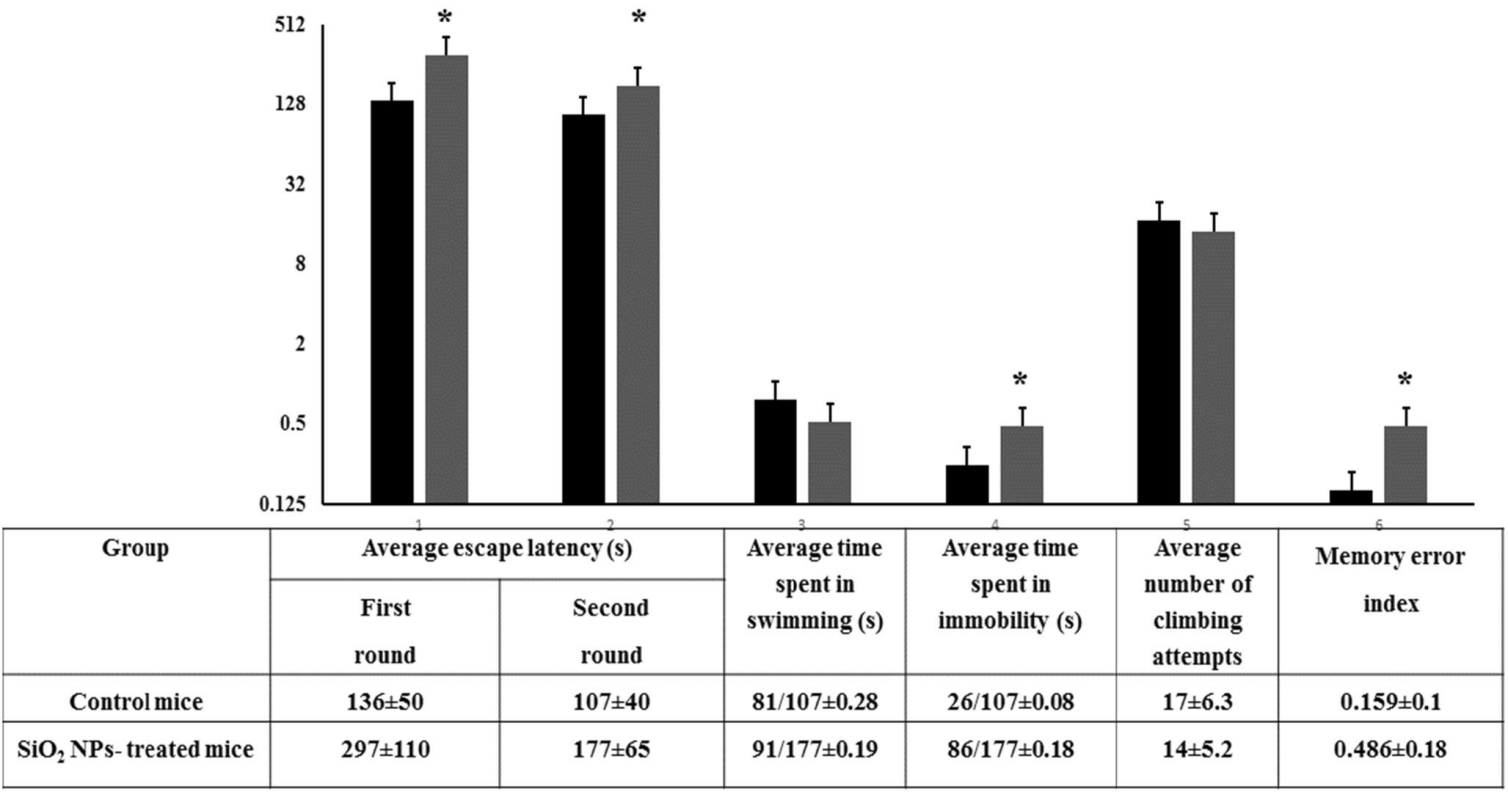
Behavioural performance of control and SiO_2_ NPs‐treated mice in Morris water‐maze test. Each mouse under study was placed in the pool to find the visible platform. The overall activity was evaluated by the following criteria: escape latency, time spent in swimming, time spent in immobility and climbing attempts number. Memory error index was calculated for all mice under study. ‘*’ indicates statistical significance using one‐way ANOVA test and *p* value < 0.05, compared with the control group. Black bar: Control mice; Grey bar: SiO_2_ NPs‐treated mice

This is standard behavioural neuroscience test used to evaluate the spatial memory and learning [45]. This test helps finding out if the hippocampus is affected or not where this brain component is involved in spatial and rational memory. As seen in Figure 12, the memory error index of the treated mice was almost three times than that of the control ones (0.486 vs. 0.159).

Hippocampus is a brain component responsible for enabling spatial memory consolidation of information and navigation. When the hippocampus suffers damage, short‐term memory loss, inability to form and retain new memories and disorientation symptoms will be early demonstrated [46]. The finding of the present behavioural test may suggest that the hippocampus damage was induced by SiO_2_ NPs affecting both memory and learning.

### 3.10 The Y‐maze test

**FIGURE 13:**
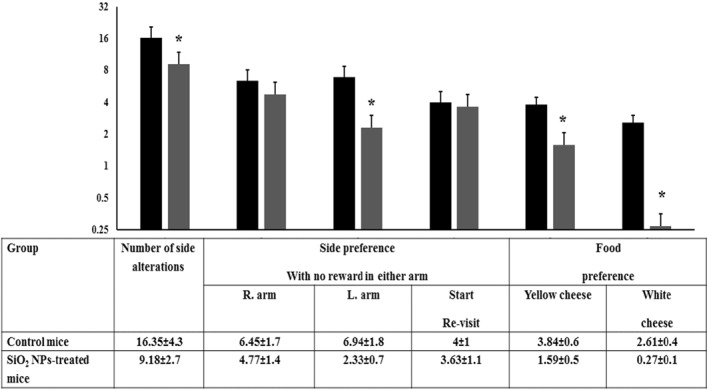
Behaviour performance exhibited by the control and SiO_2_ NPs‐treated mice in the Y‐maze test (*T* = 15 min). Each mouse under study was located individually at the base of the maze and allowed to find the food and observed for the followings: side preference, arms alterations, time spent to reach the reward and memory of reward preferences. ‘*’ indicates statistical significance using one‐way ANOVA test and *p* value < 0.05, compared with the control group. Black bar: Control mice; Gray bar: SiO_2_ NPs‐treated mice

This behavioural test is used to quantify cognitive deficits for studying spatial learning and recognition memory by letting the mouse to choose between two rewards. In addition, Y‐maze test helps in measuring the willingness of mouse to explore new environments [47]. The mouse may demonstrate inhibitory avoidance (conditioned fear) and/or one‐way escape (unconditioned fear). In this maze test, the mouse normally explores a new arm of the maze rather than returning to the previously visited one where brain components including the hippocampus, are involved in this task [47]. The decrease in number of side alteration and the number of visits to each arm with or without food reward in comparison with the control mice is an indication of cognitive deficit. As seen in Figure 13, SiO_2_ NPs‐treated mice demonstrated less number of side alterations and less preference to arms with or without reward. Accordingly, one may conclude that exposure to silica NMs can affect the cognition and the memory function.

### 3.11 The multiple T‐maze test

**FIGURE 14:**
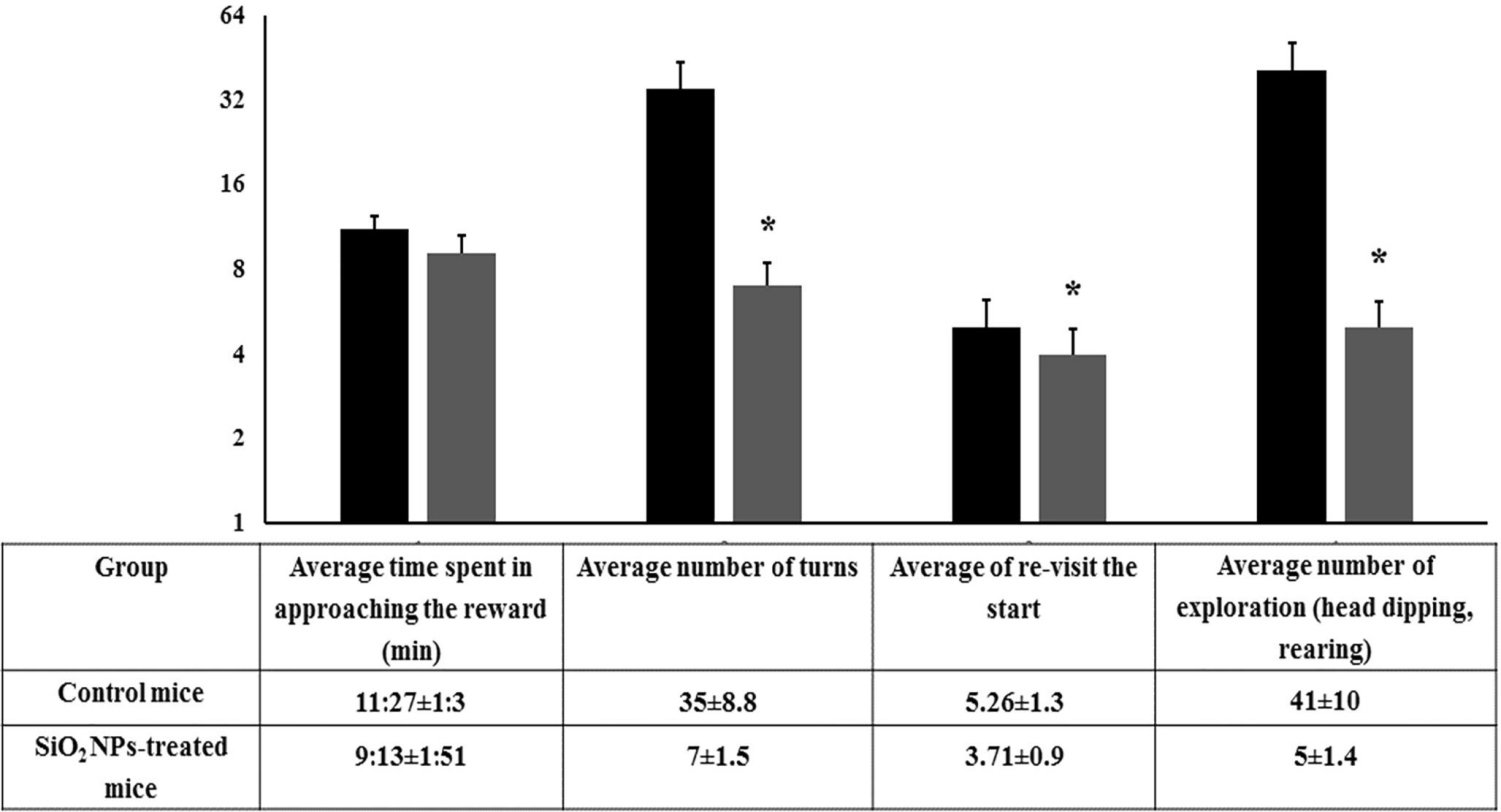
Behavioural performance of control and SiO_2_ NPs‐treated mice in the multiple T‐maze test. The food‐deprived mouse was placed individually and allowed to explore via the corridors of the maze to find food reward in the goal box. The following parameters were evaluated: The time spent in approaching the reward, number of turns, re‐visit the start, head dipping and rearing. ‘*’ indicates statistical significance using one‐way ANOVA test and *p* value < 0.05, compared with the control group. Black bar: Control mice; Grey bar: SiO_2_ NPs‐treated mice

This behavioural test is utilized in learning, memory impairment, memory formation and memory retention. This maze test is used assessing the hippocampal‐based spatial memory recognitions of maps enabling [28, 37]. This maze adds more choices, alternatives and complexity to the T‐maze and is being invested in a spatial learning assessment and in testing both the working and reference memories. As seen in Figure 14, in comparison with control mice, silica NPs‐treated mice showed more latency and less exploration to reach the goal box containing the food reward. These results demonstrate that silica NPs have the potential to modulate the nervous system leading to cognitive associated behavioural alterations.

The present findings with the context of the increasing use of silica NMs raised the concerns about their safety, benefits and the potential source of hazards to human health and the environment. The overall findings of the present work indicate that exposure to SiO_2_ NPs could induce anxiety and aggravate depression. Furthermore, the results may indicate that exposure to these NMs could decrease both locomotive and exploratory activity. Similar findings were reported by previous studies, where silica NPs aggravated depressive phenotype, inhibited exploratory behaviour and caused disturbing anxiety‐like behaviour in adult zebrafish [29]. Other toxicological studies showed that exposure to other NMs such as ZnO NPs could modulate the exploratory behaviour [37].

Some investigators considered oxidative stress as a major mechanism of silica NMs toxicity by inducing cellular components damage due to the over production of reactive oxygen species [48, 49, 50]. These psychotic alterations may be related to alterations in serotonergic and dopaminergic transmission and increased cholinergic transmission. Studies demonstrated that the hippocampus which is the major brain component involved in learning and memory storing together with other brain structures are affected by exposure to NMs [42].

## 4 CONCLUSIONS

It might be concluded from the results of the present study that exposure to silica NPs could induce behavioural alterations with indication that anxiety and depression are enhanced by subjection to these NMs. In addition, the findings indicate that subjection to these NMs could suppress locomotive and exploratory behaviours. The present work further revealed that SiO_2_ NPs have a potential neurotoxicity effect. Moreover, the present study elaborates the need for more nanotoxicological investigations to figure out the molecular mechanism of behavioural alterations of the nano‐silica. The findings may allow one to suggest more work to be carried out on the histopathological and ultrastructural alterations that might be induced by different sizes of SiO_2_ NPs in the brain components specially the hippocampus.
